# In-depth comparative analysis of malaria parasite genomes reveals protein-coding genes linked to human disease in *Plasmodium falciparum* genome

**DOI:** 10.1186/s12864-018-4654-5

**Published:** 2018-05-02

**Authors:** Xuewu Liu, Yuanyuan Wang, Jiao Liang, Luojun Wang, Na Qin, Ya Zhao, Gang Zhao

**Affiliations:** 1 Department of Pathogenic Biology, Fourth Military Medical University, Xi’an, 710032 China; 2 Department of Neurology, Xijing Hospital, Fourth Military Medical University, Xi’an, 710032 China

**Keywords:** *Plasmodium falciparum*, Virtual genome, Cerebral malaria, Parasite-infected erythrocyte surface protein 2 (PIESP2)

## Abstract

**Background:**

*Plasmodium falciparum* is the most virulent malaria parasite capable of parasitizing human erythrocytes. The identification of genes related to this capability can enhance our understanding of the molecular mechanisms underlying human malaria and lead to the development of new therapeutic strategies for malaria control. With the availability of several malaria parasite genome sequences, performing computational analysis is now a practical strategy to identify genes contributing to this disease.

**Results:**

Here, we developed and used a virtual genome method to assign 33,314 genes from three human malaria parasites, namely, *P. falciparum*, *P. knowlesi* and *P. vivax*, and three rodent malaria parasites, namely, *P. berghei*, *P. chabaudi* and *P. yoelii*, to 4605 clusters. Each cluster consisted of genes whose protein sequences were significantly similar and was considered as a virtual gene. Comparing the enriched values of all clusters in human malaria parasites with those in rodent malaria parasites revealed 115 *P. falciparum* genes putatively responsible for parasitizing human erythrocytes. These genes are mainly located in the chromosome internal regions and participate in many biological processes, including membrane protein trafficking and thiamine biosynthesis. Meanwhile, 289 *P. berghei* genes were included in the rodent parasite-enriched clusters. Most are located in subtelomeric regions and encode erythrocyte surface proteins. Comparing cluster values in *P. falciparum* with those in *P. vivax* and *P. knowlesi* revealed 493 candidate genes linked to virulence. Some of them encode proteins present on the erythrocyte surface and participate in cytoadhesion, virulence factor trafficking, or erythrocyte invasion, but many genes with unknown function were also identified. Cerebral malaria is characterized by accumulation of infected erythrocytes at trophozoite stage in brain microvascular. To discover cerebral malaria-related genes, fast Fourier transformation (FFT) was introduced to extract genes highly transcribed at the trophozoite stage. Finally, 55 candidate genes were identified. Considering that parasite-infected erythrocyte surface protein 2 (PIESP2) contains gap-junction-related Neuromodulin_N domain and that anti-PIESP2 might provide protection against malaria, we chose PIESP2 for further experimental study.

**Conclusions:**

Our analysis revealed a limited number of genes linked to human disease in *P. falciparum* genome. These genes could be interesting targets for further functional characterization.

**Electronic supplementary material:**

The online version of this article (10.1186/s12864-018-4654-5) contains supplementary material, which is available to authorized users.

## Background

Malaria is still a major global public health problem. According to the World Malaria Report 2016, more than 200 million people suffer from malaria and over 400,000 people die as a consequence of this disease [1]. Malaria is caused by parasitic protozoans belonging to the genus *Plasmodium*. At least five species of *Plasmodium* are capable of infecting humans, including *P. falciparum*, *P. knowlesi*, *P. vivax*, *P. ovale*, and *P. malariae* [2]. Among them, *P. falciparum* causes the most-often fatal and medically severe form of the disease, and has thus received the most attention. The animal malaria parasites, such as *P. berghei*, *P. chabaudi*, *P. vinckei*, and *P. yoelii*, are natural parasites of rodents. They are usually used as models to study malarial infections in the laboratory [3].

Two biological features of *P. falciparum* are particularly noteworthy regarding its ability to cause human disease. One is that, as a human malaria parasite, *P. falciparum* can invade and parasitize human erythrocytes, while the rodent malaria parasites are infectious to rodent species but not humans, suggesting that *P. falciparum* possesses some properties required for parasitizing human erythrocytes. The other feature is that *P. falciparum* is much more virulent than all other human malaria species. *P. falciparum* infection may progress to severe malaria, which manifests as one or more of the following severe complications: cerebral malaria (CM), severe malaria anemia, and acidosis/respiratory distress (RD) [4]. Among these complications, CM accounts for a significant proportion of malaria-related deaths and shows potential for the induction of neurological deficits in survivors [5]. It is characterized by the accumulation of *P. falciparum*-infected RBCs (iRBCs) at the pigmented trophozoite stage in the microvasculature of the brain [6]. Very few malaria deaths have been reported for *P. vivax* and *P. knowlesi*. In fact, *P. vivax* rarely kills the infected individual and is responsible for most cases of benign tertian malaria [7]. Identification of the genetic basis of the aforementioned biological features can help in the discovery of genes contributing to human disease, the development of new strategies to prevent *P. falciparum* infecting humans, and the treatment of severe malaria in humans.

Recently, the genome sequences of several malaria parasites have become publicly available [8], making comparative genome analysis a practical strategy to search for human disease-related genes. A series of genes contributing to human disease have been identified by this method. For example, the comparative analysis of human and rodent malaria parasite genomes revealed that two enzymes, PF3D7_0520500 and PF3D7_0614000, which are essential enzymes in thiamine biosynthesis, are absent in rodent malaria parasites [9]. As the elimination of thiamine greatly impairs the erythrocytic multiplication rates of malaria parasites, the presence of the thiamine synthesis pathway in human malaria parasites can be seen as an adaption to increase the viability of such parasites in human erythrocytes and contribute to human pathogenesis. Furthermore, a comparison of the genome of non-cytoadherent *P. falciparum* D10 to that of cytoadherent *P. falciparum* 3D7 revealed a subtelomeric deletion on the right arm of chromosome 9 in D10 [10]. Further experimental study of 25 genes in this subtelomeric region indicated that the absence of virulence-associated protein 1 (*Pf*VAP*1*) was responsible for the non-cytoadherent phenotype of D10, demonstrating that *Pf*VAP1 is a virulence-related factor [11]. Although comparative genome analysis is feasible for the identification of genes associated with a particular phenotype, there were two limitations in previous analyses: First, earlier analyses only focused on genes specific to a group of species (group-specific), while genes conserved across all species but expanding in a group of species (group-expansion) were usually not considered. Second, using the previous method to identify the species-enriched genes among *n* species required at least $\left(\genfrac{}{}{0pt}{}{n}{2}\right)$ comparisons, which makes the task quite resource-intensive when *n* is too large.

In this study, to identify genes related to human disease in the *P. falciparum* genome, we developed a virtual genome method that overcomes the aforementioned limitations. Three human malaria parasites, namely, *P. falciparum*, *P. knowlesi* and *P. vivax*, and three rodent malaria parasites, namely, *P. berghei*, *P. chabaudi* and *P. yoelii*, were selected because these species have NCBI taxon IDs, and their host-tropism and virulence are relatively well characterized. We hypothesized that all of the analyzed malaria parasites had a common virtual genome, where each virtual gene actually represents a cluster of real genes whose protein sequences are similar. The phenotypic difference can be attributed to differences in the expression of virtual genes. Genes associated with a particular biological feature are those highly or specifically expressed in the group of species with such features. To look for genes linked to human disease, first, we established a protein sequence similarity network through sequence alignment and utilized the modularity method to partition this network into thousands of clusters. The obtained clusters varied in terms of the number of genes, ranging from one to more than 1000 genes. Each cluster was considered a virtual gene. Second, we compared the enriched values of all clusters in human malaria parasites with those in rodent malaria parasites to find genes responsible for *P. falciparum* parasitizing human erythrocytes. Third, we looked for genes related to virulence by comparing cluster values in *P. falciparum* with those in *P. vivax* and *P. knowlesi*. Finally, to discover novel molecules contributing to CM, we integrated gene expression data and extracted virulence-related genes highly transcribed at the trophozoite stage. One candidate gene was selected as an attractive starting point for follow-up experimental investigation.

## Results

### Establishment of virtual genome method by sequence cluster identification

**Fig. 1:**
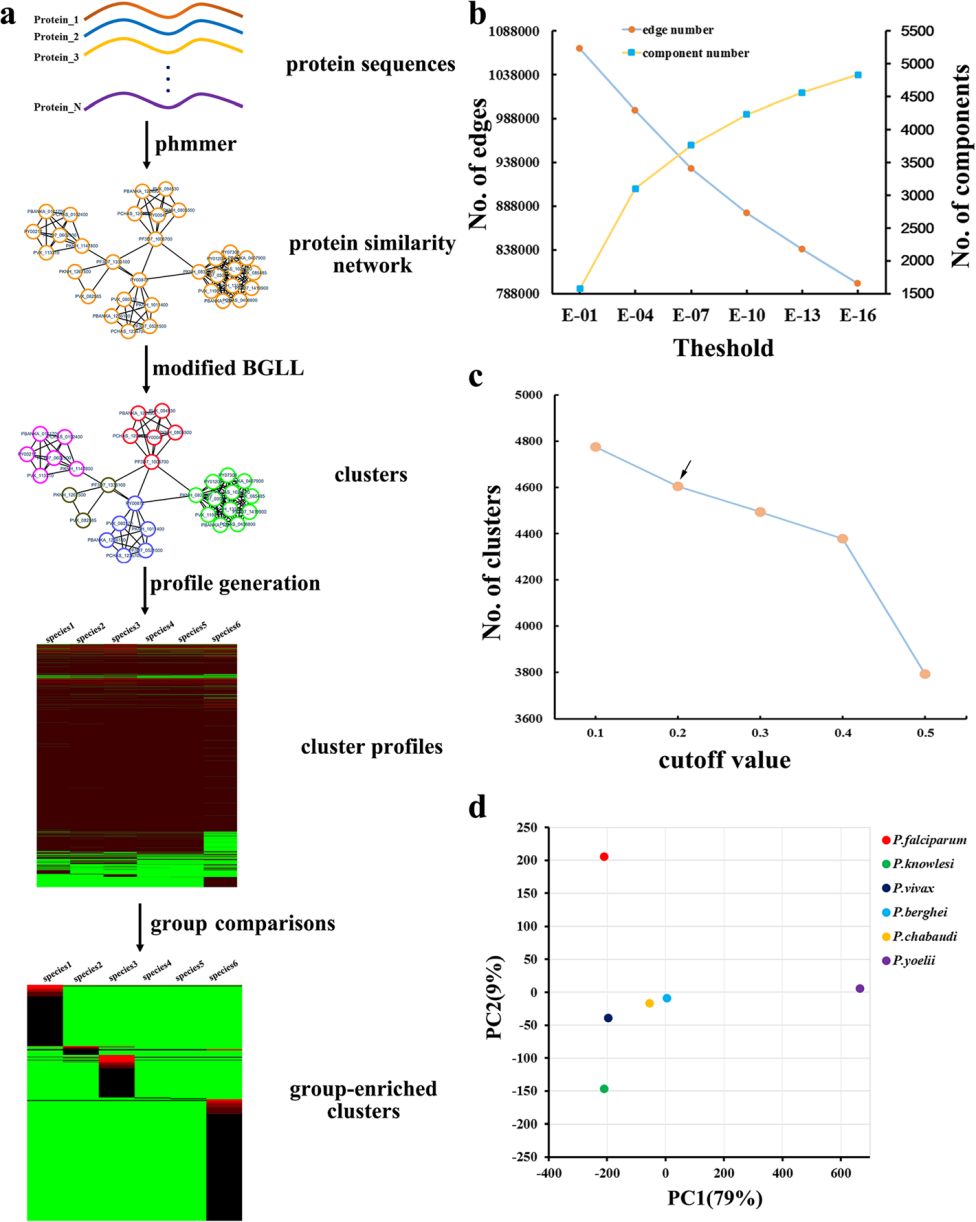
Identification of group-enriched genes by virtual genome method. **a** Workflow of our comparative analysis. Protein sequence alignment was performed using phmmer to construct a protein similarity network where each edge represents a significant hit between query and target. Then, a modified BGLL algorithm was applied to find clusters within this network. Each cluster was considered as a virtual gene. Genes within these clusters were allocated to the species from which they originated, subsequently generating enriched values of all clusters in six species. Group-enriched genes can be identified by comparing cluster values in ingroup species with those in outgroup species. **b** The number of edges and the number of components included in the protein similarity networks that were obtained under different thresholds. **c** The number of clusters identified by the modified BGLL algorithm using different cut-off values of modularity. The arrow indicates the cut-off value used in this study. **d** Principal component analyses (PCA) of the enriched values of all clusters in six *Plasmodium* species. Components 1 (PC1) and 2 (PC2) represent 79% and 9% of total variance, respectively

*P. falciparum* can parasitize human erythrocytes and is the most virulent malaria parasite. To identify the genetic basis of these important biological features, we performed comparative analysis of three human and three rodent malaria parasite genomes. We assumed that all of these *Plasmodium* species have a common virtual genome, but differ in virtual gene expression. Genes highly expressed in a subgroup of *Plasmodium* species are frequently associated with the unique feature of such parasites. For example, *var.* is specific to *P. falciparum*. It encodes the prime virulence factor PfEMP1 involved in the attachment of infected erythrocytes to microvascular [12]. To find species-group enriched genes, we performed protein sequence alignment to construct a network where each edge represents a significant hit between query and target (Fig. 1a). Then, we developed a modified BGLL (see methods) algorithm and applied it to identify sequence clusters within this network. Genes within each cluster are significantly similar in their protein sequences. Finally, the members of each cluster are allocated to the *Plasmodium* species from which they are derived, generating enriched values of all clusters in those species. The enriched value of a cluster can be considered to reflect the expression level of such a cluster. Species group-enriched clusters can be found by comparing the cluster values in all ingroup species with those in outgroup species. Genes within the enriched clusters are then defined as species group-enriched genes.

Each protein sequence of the six *Plasmodium* species was used as a query and searched against the total protein sequences of these species by phmmer. Figure 1b shows the numbers of edges and components within the disconnected network using expectation values ranging from 1E − 1 to 1E − 16. Although the relationship between the number of edges and the threshold values was almost linear, an unapparent knee point was still observed at 1E − 7. The number of components significantly increased at 1E − 4 and mildly increased at 1E − 7. Further decrease in the threshold led to a slight increase in the number of components. Therefore, we set the threshold as 1E − 7. The resulting disconnected network consisted of 931,335 edges and 3768 components. We then adopted a modified BGLL algorithm to identify sequence clusters within this disconnected network (see Methods). Figure 1c shows the number of clusters identified using different modularity cut-off values. An increase in cut-off values from 0.4 to 0.5 led to a significant drop in the number of clusters, implying that many cluster structures were not well identified. The number of clusters had a relative apparent increase when the cut-off value was reduced from 0.2 to 0.1, indicating that several homologs had been classified into different groups. To avoid the presence of a supercluster consisting of several independent clusters and the misclassification of remote homologs, we set the cut-off value to 0.2. Under this condition, 33,314 genes were grouped into 4605 clusters (Additional file 1: Table S1). Thus, we achieved a virtual genome which represents a collection of 4605 virtual genes.

Among the obtained clusters, some of them, such as Cluster_12 and Cluster_223 consisting of 4 and 9 genes, respectively, comprise genes from a single *Plasmodium* species. Some clusters, such as Cluster_15 and Cluster_16 which contain 39 and 19 genes, include genes from six species (Additional file 2: Figure S1), respectively. Cluster_1 has the largest number of members, and it contains 1096 vertices. The genes within this cluster were all from rodent malaria parasite genomes, but absent in human malaria parasites. A total of 426 clusters constitute a member. On the basis of the obtained sequence clusters, we generated the expression profiles of all clusters in six *Plasmodium* species, and each column shows the enrichment values of all clusters in such a species (Additional file 3: Table S2). Principal component analysis (PCA) of all of these cluster values demonstrated that *P. falciparum* differs from five other species in the second component, which represents 9% of the variance, while *P. yoelii* differs from other parasites in the first component, which represents 79% of the variance (Fig. 1d). Comparison of the cluster profiles of six *Plasmodium* species can reveal species group-enriched genes, including group-specific genes and group-expansion genes. For example, Cluster_32 is composed of 227 genes and found to be unique to *P. falciparum*. Genes within this cluster encode RIFIN/STEVOR proteins which exist specifically in *P. falciparum* [9]. Additionally, Cluster_161, which consists of 27 *FIKK* genes, was found in all species, but was much more abundant in *P. falciparum* than in all other *Plasmodium* species, consistent with the report that the *FIKK* gene had been amplified in *P. falciparum* to approximately 20 sequence-related members [13]. Therefore, our method was shown to be feasible for identifying species group-enriched genes.

In comparison with previous genomic analysis where comparison was performed between any two of these species [9], our method outperforms this in two aspects. First, our analysis is more comprehensive than the previous approach. In our analysis, we can identify both group-specific and group-expansion genes, while in the previous comparative analysis, the investigators usually only focused on group-specific genes. Second, our method makes the identification of genes underlying phenotypic differences much simpler than the previous analysis because we avoid performing comparative analysis of all pairs of species. Thereafter, we looked for *P. falciparum* genes linked to the infection of human erythrocytes and virulence by our method. A cluster was considered to be enriched in a group of species if its minimal value in all ingroup species was fivefold higher than its maximal value in the outgroup species.

### Identification of *P. falciparum* genes responsible for parasitizing human erythrocytes

**Fig. 2:**
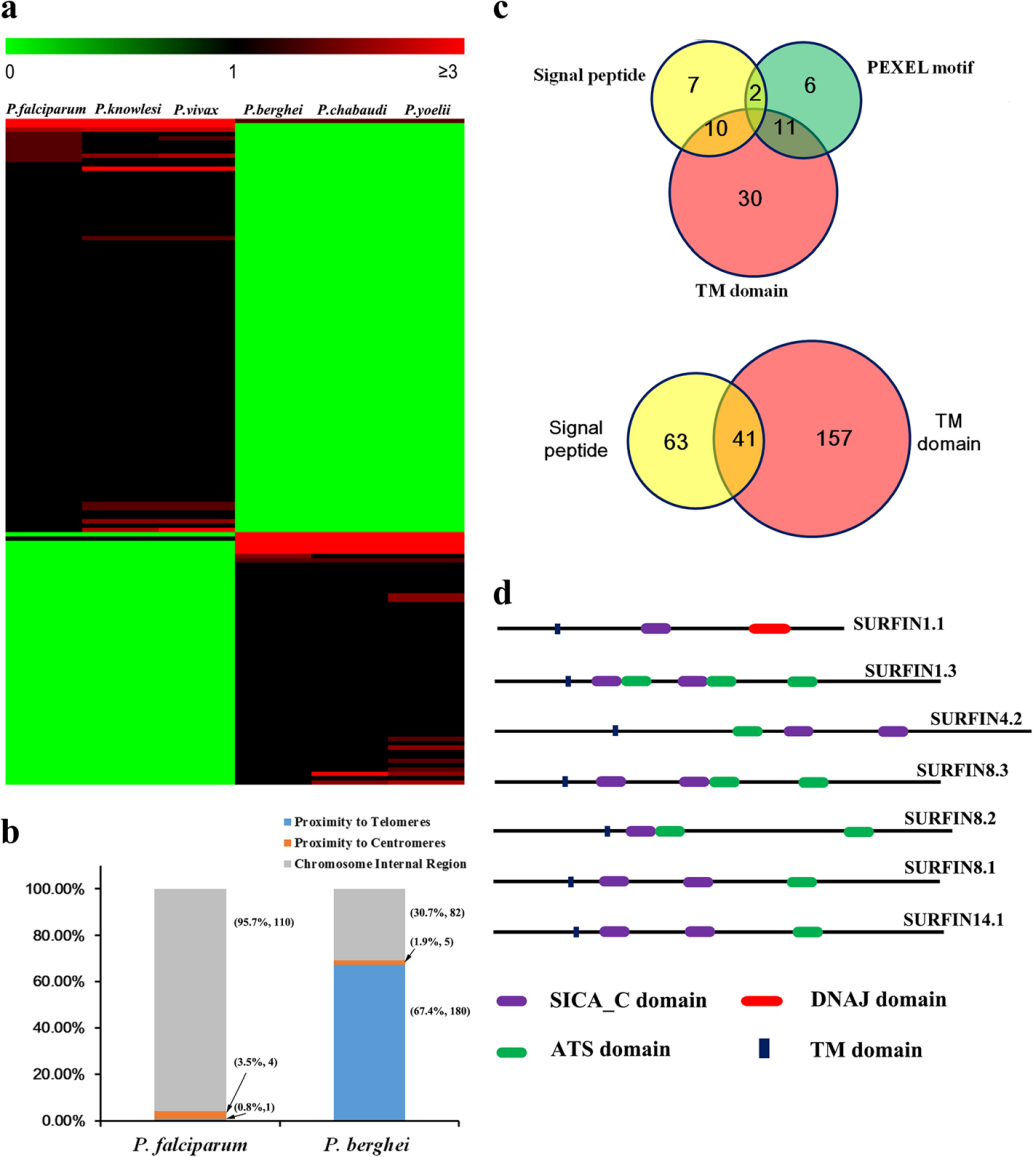
Identification of *P. falciparum* genes responsible for parasitizing human erythrocytes. **a** Heat map showing the clusters enriched in human and malaria parasites. Green, black, and red indicate cluster values equal to zero, one, and higher than one, respectively. **b** Bar plot displaying the genomic location of 115 *P. falciparum* genes and 267 *P. berghei* genes. Proximity to telomeres and proximity to centromeres refer to the genome regions within 40 kb away from telomeres and 10 kb away from centromeres, respectively. The rest of the genome was referred to as the chromosome internal region. The numbers in each parenthesis represent the number of genes and the percentage to human or malaria enriched genes. **c** Venn diagram showing the number of *P. falciparum* genes (upper panel) or *P. berghei* genes (lower panel) whose proteins contain a signal peptide, a transmembrane domain, or a PEXEL motif. **d** Domain models of SURFIN family members. Domains were identified through CD-search (https://www.ncbi.nlm.nih.gov/Structure/cdd/wrpsb.cgi) with a cut-off value of 0.01. TM domain stands for transmembrane domain

As a human malaria parasite, *P. falciparum* can infect human erythrocytes but not the erythrocytes of rodent species, while the rodent malaria parasites are incapable of parasitizing human erythrocytes, suggesting that the *P. falciparum* genes enriched in human malaria parasites might be required for parasitizing human erythrocytes. To identify genes linked to this biological feature, we compared the enriched values of all clusters in human malaria parasites with those in rodent malaria parasites. As shown in Fig. 2a, there were 94 and 57 clusters enriched in human and rodent malaria parasites, respectively. To illustrate the difference between human and rodent malaria parasites in detail, *P. falciparum* genes within human-enriched clusters were compared with *P. berghei* genes included in rodent-enriched clusters. In total, 121 *P. falciparum* genes and 398 *P. berghei* genes were identified. After removing pseudogenes, 115 *P. falciparum* genes and 289 *P. berghei* genes were retained for further analysis (Additional file 4: Table S3 and Additional file 5: Table S4).

Genomic location analysis revealed that very few of these *P. falciparum* genes are located in the vicinity of the telomeres or centromeres, but almost all of them are located in the chromosome internal regions (Fig. 2b and Additional file 6: Figure S2), while 180 of 267 *P. berghei* genes with a known location are present in the subtelomeric regions and five genes are located in proximity to the centromeres (Fig. 2b and Additional file 7: Figure S3), demonstrating that human and rodent parasite-enriched genes have different chromosome locations. Sequence feature analysis of proteins encoded by these genes indicated that approximately 10% (28/289) of *P. berghei* candidate genes encode intracellular proteins, significantly less than that of *P. falciparum* candidate genes, for which the rate is about 44%. In *P. falciparum*, there were 51 transmembrane domain-containing proteins, 10 of which have a signal peptide and 11 of which contain a PEXEL motif (Fig. 2c upper panel). Meanwhile, in *P. berghei*, there were 198 proteins containing a transmembrane domain, mostly because of the presence of the plasmodium interspersed repeat (*PIR*) multigene gene family, whose proteins were displayed on the surface of infected erythrocytes [14]. Nearly one-fifth (41/198) of them possess signal peptides, but none of them has a canonical PEXEL motif (Fig. 2c lower panel).

Among the human parasite-enriched clusters, Cluster_99 was the sole cluster that consisted of group-expansion genes. This cluster comprised genes from the *PHISTc* gene family, which is a subtype of the PHIST family [15]. This family was found to be amplified in human malaria parasites to more than 10 members, but has only a few members in the rodent malaria parasites (see Additional file 3: Table S2). A recent study showed that a PHISTc protein, named PFI1780w, localizes underneath the membrane of infected erythrocytes and participates in the remodeling of host erythrocytes by interacting with the ATS (acidic terminal segments) domain of *P. falciparum* erythrocyte membrane protein 1 (PfEMP1) [16]. Apart from Cluster_99, the remaining clusters were specific to human malaria parasites. Of them, Cluster_13 was the largest cluster, comprising seven members of the *SURF* gene (surface-associated interspersed gene) family. Apart from the SURFIN1.1 protein whose intracellular region contains a SICA_C (schizont-infected cell agglutination C-terminal) domain and a DNAJ domain, all other SURFIN proteins are characterized by one or two SICA_C domains and one to three ATS domains (Fig. 2d left panel). SURFIN4.2 is the best characterized member. It can interact with F-actin and spectrin through its internal domain and be co-transported with PfEMP1 and RIFIN to the surface of infected erythrocytes [17, 18]. Analysis of the expression of *SURF* members revealed that SURFIN4.2 was highly transcribed at the ring stage, while SURFIN8.1, 8.2, 8.3, 1.3, and 14.1 were maximally expressed at the trophozoite stage (Additional file 8: Figure S4). Very low expression of SURFIN1.1 was observed. This difference in expression dynamics implied that these members might play different roles in the intraerythrocytic developmental cycle of the *P. falciparum* parasite. Besides *SURF* genes, two group-specific genes, *PF3D7_0520500* and *PF3D7_0614000*, which are required for thiamine biosynthesis, were found only to be present in human malaria parasites, but not in rodent malaria parasites (see Additional file 4: Table S3). This is in agreement with a previous report describing that the thiamine biosynthesis pathway was absent in rodent malaria parasites [9].

Apart from the aforementioned genes, there were many additional protein-coding genes specific to human malaria parasites. Proteins encoded by *PF3D7_0731100*, *PF3D7_1002100*, and *PF3D7_1302000* play a role in the increasing rigidity and adhesiveness of infected erythrocytes by trafficking and displaying PfEMP1 on the host erythrocytes [19]. *PF3D7_1322100* is a histone-lysine N-methyltransferase gene and its protein product methylates histone H3K36 and plays a role in immune evasion [20, 21]. *PF3D7_0807700* encodes a serine protease, DegP, which has a role in the growth and development of *P. falciparum* through its ability to confer protection against thermal/oxidative stress [22]. *PF3D7_1206100* encodes an IMP-specific 5′-nucleotidase, which is involved in purine metabolism. However, the functions of approximately 44% (51/115) of human malaria parasite-enriched genes are unknown. Taking these findings together, genes enriched in human-specific malaria parasites are related to a variety of biological processes and the combination of these genes might be responsible for the overall ability of *P. falciparum* to parasitize human erythrocytes.

### Identification of genes related to the virulence of *P. falciparum*

**Fig. 3:**
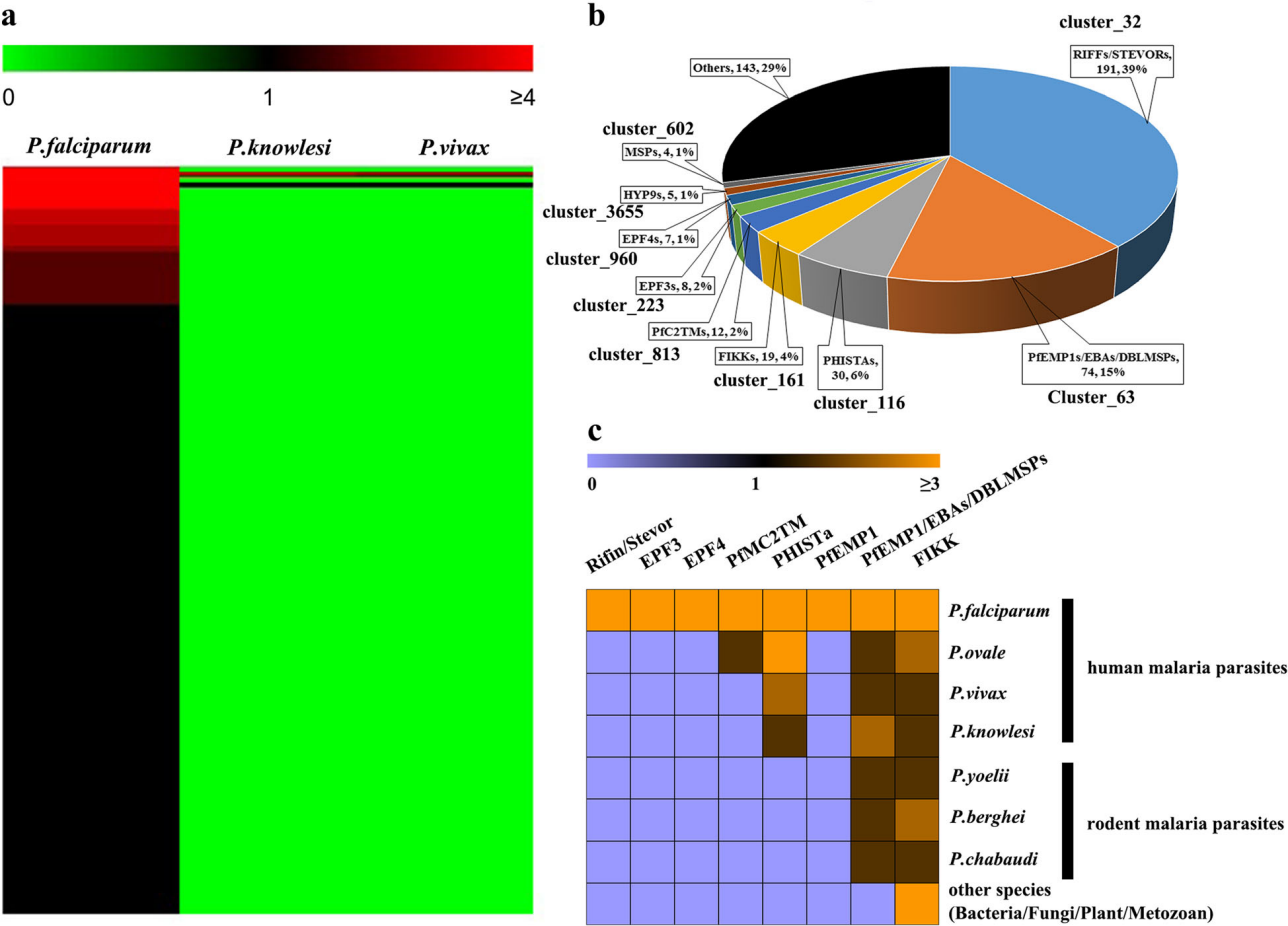
Candidate genes related to virulence of the *P. falciparum* parasite. **a** Heat map showing the clusters enriched in *P. falciparum*. Green, black, and red indicate cluster values equal to zero, one, and higher than one, respectively. **b** Pie chart displaying the enrichment of each cluster in candidate genes. The numbers in each box represent the cluster size and the percentage to the total number of *P. falciparum* enriched genes. **c** Heat map showing the number of members detected in *Plasmodium* species or other species using hidden Markov models of seven families. Deep purple indicates no member was found, black indicates one member was detected, and gold indicates more than one member was discovered

*P. falciparum* is much more virulent than any other human malaria parasites. We looked for genes linked to virulence by comparing the cluster profile in *P. falciparum* with those in *P. vivax* and *P. knowlesi*. As shown in Fig. 3a, there were 141 *P. falciparum*-enriched clusters, of which 139 were unique to *P. falciparum*. After removing 114 pseudogenes, the remaining 493 genes were analyzed further (Additional file 9: Table S5). Gene Ontology (GO) subcellular localization analysis demonstrated that protein products of these genes were enriched in the infected host cell surface knob, host cell membrane, and Maurer’s cleft (Table 1), suggesting their possible roles in cell–cell adhesion. Additionally, biological process analysis revealed that these genes were associated with the regulation of cell adhesion and erythrocyte aggregation (Table 2).

**Table 1:** Cellular component analysis of proteins produced by virulence-related candidate genes. Enriched terms were ranked according to their percentage of background. The top 20 terms are listed

ID	Name	Background count	Result count	Percent of background	Bonferroni adjusted *P*-value
GO:0020030	infected host cell surface knob	54	51	94.4	1.71E-26
GO:0020002	host cell plasma membrane	217	201	92.6	5.58E-118
GO:0033644	host cell membrane	230	205	89.1	1.39E-118
GO:0044218	other organism cell membrane	230	205	89.1	1.39E-118
GO:0044279	other organism membrane	230	205	89.1	1.39E-118
GO:0020036	Maurer’s cleft	229	182	79.5	8.18E-97
GO:0033655	host cell cytoplasm part	367	243	66.2	4.01E-125
GO:0020003	symbiont-containing vacuole	184	117	63.6	6.25E-51
GO:0065010	extracellular membrane-bounded organelle	184	117	63.6	6.25E-51
GO:0033643	host cell part	472	298	63.1	2.25E-161
GO:0043230	extracellular organelle	186	117	62.9	1.45E-50
GO:0030430	host cell cytoplasm	398	250	62.8	7.23E-126
GO:0033646	host intracellular part	398	250	62.8	7.23E-126
GO:0043656	intracellular region of host	399	250	62.7	1.13E-125
GO:0043245	extraorganismal space	479	298	62.2	5.47E-160
GO:0018995	host	479	298	62.2	5.47E-160
GO:0043657	host cell	479	298	62.2	5.47E-160
GO:0044217	other organism part	479	298	62.2	5.47E-160
GO:0044216	other organism cell	479	298	62.2	5.47E-160
GO:0044215	other organism	479	298	62.2	5.47E-160

**Table 2:** Biological process analysis of proteins produced by virulence-related candidate genes. Enriched terms were ranked according to their percentage of background. The top 20 terms are listed

ID	Name	Background count	Result count	Percent of background	Bonferroni adjusted *P*-value
GO:0034110	regulation of homotypic cell-cell adhesion	190	190	100	3.94E-114
GO:0022407	regulation of cell-cell adhesion	190	190	100	3.94E-114
GO:0034118	regulation of erythrocyte aggregation	190	190	100	3.94E-114
GO:0020013	modulation by symbiont of host erythrocyte aggregation	190	190	100	3.94E-114
GO:0030155	regulation of cell adhesion	190	190	100	3.94E-114
GO:0044068	modulation by symbiont of host cellular process	192	191	99.5	1.44E-114
GO:0044003	modification by symbiont of host morphology or physiology	194	191	98.5	5.12E-114
GO:0051817	modification of morphology or physiology of other organism involved in symbiotic interaction	194	191	98.5	5.12E-114
GO:0051809	passive evasion of immune response of other organism involved in symbiotic interaction	204	200	98	2.96E-120
GO:0020033	antigenic variation	204	200	98	2.96E-120
GO:0035821	modification of morphology or physiology of other organism	195	191	97.9	9.62E-114
GO:0020035	cytoadherence to microvasculature, mediated by symbiont protein	164	160	97.6	2.98E-92
GO:0044406	adhesion of symbiont to host	164	160	97.6	2.98E-92
GO:0051834	evasion or tolerance of defenses of other organism involved in symbiotic interaction	206	200	97.1	1.04E-119
GO:0052173	response to defenses of other organism involved in symbiotic interaction	206	200	97.1	1.04E-119
GO:0051832	avoidance of defenses of other organism involved in symbiotic interaction	206	200	97.1	1.04E-119
GO:0051707	response to other organism	206	200	97.1	1.04E-119
GO:0043207	response to external biotic stimulus	206	200	97.1	1.04E-119
GO:0051805	evasion or tolerance of immune response of other organism involved in symbiotic interaction	206	200	97.1	1.04E-119
GO:0051807	evasion or tolerance of defense response of other organism involved in symbiotic interaction	206	200	97.1	1.04E-119

Figure 3b shows the proportion of each cluster among these candidate genes. We focused on the clusters containing more than five members. Of these clusters, Cluster_63 and Cluster_161 were composed of group-expansion genes. Cluster_63 mainly comprised *var.* gene family members, which encode the prime virulence factor PfEMP1. The extracellular region of PfEMP1 contains DBL (Duffy binding-like) and CIDR (cysteine-rich inter-domain region) domains. The DBL domain can bind intercellular adhesion molecule 1 (ICAM1) and the CIDR domain can bind endothelial protein C receptor (EPCR) or CD36 on the endothelium surface [12, 23]. By interacting with erythrocyte surface proteins, PfEMP1 mediates the attachment of infected erythrocytes to the endothelium, subsequently resulting in CM. Cluster_161 was composed of the *FIKK* gene family. This family encodes protein kinases that co-localize with Maurer’s cleft proteins and have a role in remodeling of the erythrocyte surface [13]. Apart from the above two clusters, all of the remaining five clusters were specific to *P. falciparum*. The largest cluster of them consists of *rif/stevor* gene family members. Protein products of this family are expressed on the surface of infected RBCs where they bind these cells together to form large rosettes or microvascular endothelial cells, subsequently leading to the occurrence of severe malaria [17, 24]. The second largest group contains *PHISTa* family members. The transcription of several of them was found to be induced under febrile conditions [25]. As PHISTa proteins contain the PEXEL motif and a transmembrane domain close to their N-terminus, in a febrile state, they might be exported to the host membrane and involved in interacting with host cells. The remaining three clusters were the *PfMC2TM*, *EPF3*, and *EPF4* gene families. Proteins encoded by these families are exported to Maurer’s clefts, which act as a platform for marshaling exported parasite proteins addressed to the host cell plasma membrane or displayed on the erythrocyte surface, implying their possible role in assisting the correct presentation of membrane proteins on the surface of infected erythrocytes [26, 27].

To identify possible members of the above seven families in other genome-sequenced organisms, the profile hidden Markov model of each cluster was built and used as a query for a search against the reference proteome database. Except for the FIKK family members found in other species, such as species of bacteria, fungi, and plants, the remaining six gene families were only found in the *Plasmodium* genus (Fig. 3c). In particular, the RIFIN/STEVOR, EPF3, and EPF4 families were unique to *P. falciparum*, and the PfMC2TM and PHISTa families were found only in human malaria parasites. The PfEMP1/EBAs/DBLMSP family, all members of which contain a DBL domain, comprises proteins from the EMP1, EBA, and DBLMSP families. This family has nearly 80 members in *P. falciparum* and a few members in other *Plasmodium* species, but no members of it were detected in other organisms, suggesting that this family arose in the *Plasmodium* genus and then underwent dramatic proliferation in *P. falciparum*. However, after removing EBA and DBLMSP family members, we established a new profile hidden Markov model for PfEMP1 proteins. Searching the reference database using this new model demonstrated that the PfEMP1 family exists only in *P. falciparum* (Fig. 3c). Thus, although the DBL domain can be found in all six species, PfEMP1 proteins are unique to *P. falciparum* and were amplified in this species. Additionally, in this new model, we identified a conserved peptide region harbored in DBL-1α domain of all PfEMP1 proteins (Additional file 10: Figure S5), implying that antibody recognizing this region might elicit cross-reactive response to a substantial number of PfEMP1 variants.

The remaining clusters specifically belong to *P. falciparum*. Proteins of several genes within these clusters have been well characterized. These include the reticulocyte binding protein homologue 5 (RH5), which aids parasite invasion of erythrocytes by binding CD147 on the erythrocyte surface [28], and two membrane protein trafficking molecules, PF3D7_0730900 and PF3D7_1478600, which play a role in trafficking and display of the virulence protein PfEMP1 on the host erythrocytes; disruption of these genes leads to no or very low levels of surface-expressed PfEMP1 [19], as well as a merozoite surface protein 2 (MSP2), which is involved in fibril formation [29]. In addition, histidine-rich protein II (HRPII) released by erythrocytes infected with *P. falciparum* can inhibit antithrombin. It binds cellular glycosaminoglycans and prevents their interaction with antithrombin, thereby contributing to the procoagulant state associated with *P. falciparum* infection [30]. However, for nearly one-quarter of *P. falciparum*-enriched genes, the function is unknown, so this requires further elucidation. Taking these findings together, the majority of *P. falciparum*-enriched genes encode exported or membrane-associated proteins that either serve as adhesins or participate in membrane protein trafficking, erythrocyte invasion, and the inhibition of antithrombin, pointing towards to the virulence of the *P. falciparum* parasite.

### Identification of novel molecules contributing to cerebral malaria

CM is the most life-threatening complication of human malaria. Many parasite proteins that mediate the binding of infected erythrocytes to endothelium remain unknown, impeding our understanding of the molecular mechanisms behind CM. To identify novel genes potentially related to CM, we performed sequence feature analysis of *P. falciparum*-enriched genes and identified 308 genes whose proteins contain transmembrane domains. Genes whose products were annotated as peripheral or integral proteins of the Maurer’s cleft membrane were removed, including members of the *EPF4*, *PfMC2TM*, and *FIKK* families. Genes producing proteins associated with membrane protein trafficking were also removed. Three genes, namely, *PF3D7_1431800*, *PF3D7_0529200*, and *PF3D7_1140000*, encode proteins annotated as apyrase, sugar transporter, and carbonic anhydrase, respectively. They are unlikely to serve as adhesion proteins and were thus not considered further. Finally, we identified a total of 279 candidate genes that may contribute to CM.

**Fig. 4:**
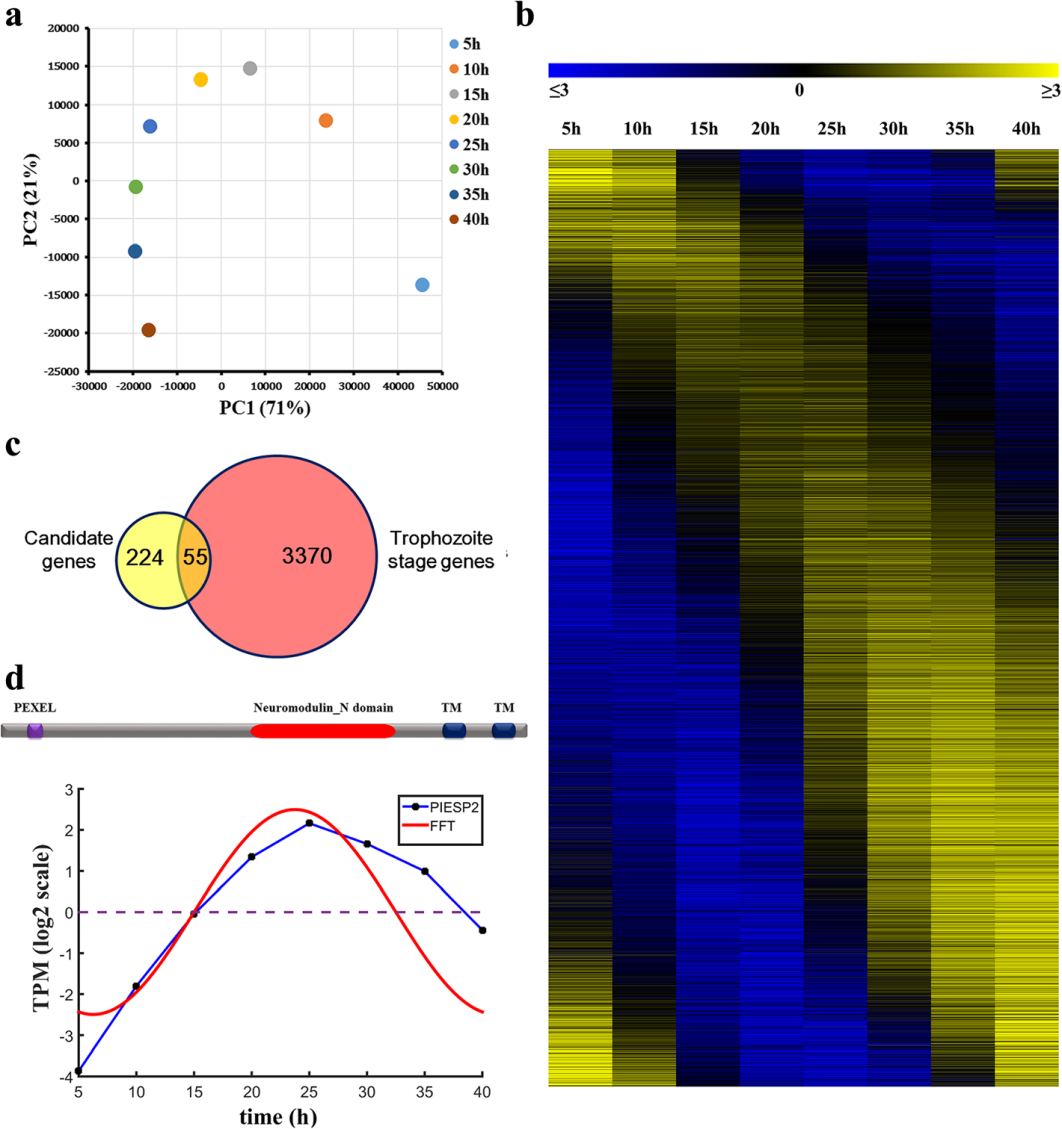
Identification of *P. falciparum* genes contributing to cerebral malaria. **a** Principal component analysis performed on eight RNA-seq datasets. Components 1 (PC1) and 2 (PC2) represent 71% and 21% of total variance, respectively. Datasets of two adjacent time points tend to be located close together within the plot. **b** The periodic genes identified by FFT ordered by the time points of their peak expression. Expression values of each transcript were log2-scaled and centered by subtracting their mean value. **c** Venn diagram of the number of genes transcribed at the trophozoite stage and that of candidate genes whose proteins contain transmembrane domains. **d** Domain model of PIESP2 protein (upper panel) and expression signals of PIESP2 in the intraerythrocytic cycle (lower panel). TM represents transmembrane domain. Blue line represents the observed expression level of PIESP2 and red line is the fitting curve using FFT

Not all of the candidate genes are associated with CM because some genes were not expressed at the trophozoite stage. To improve our analysis, we thus needed to integrate gene expression information into our analysis. The RNA-seq dataset, GSE23787, which features gene expression data measured during the intraerythrocytic development cycle of *P. falciparum*, was adopted to identify genes highly expressed at the trophozoite stage. PCA analysis revealed that expression datasets of two adjacent time points tend to be closer together in the PCA plot (Fig. 4a), suggesting a small difference between them. However, the distance in the plot between the datasets from 5 and 10 h was larger than that of any other two adjacent time points, demonstrating that the *P. falciparum* parasite experienced a clear change in gene expression at 10 h. A previous study revealed that genes induced in this stage are mainly associated with cytoplasmic transcriptional and translational machinery, glycolysis and ribonucleotide biosynthesis [31]. FFT was thus introduced to extract genes associated with the trophozoite stage. The amplitude of expression of each gene was computed. We only retained expression signals with maximal amplitude at frequency *ω* = 1. After removing genes with mean of log2 transformed TPM < 2 or amplitude A < 0.5 at *ω* = 1, the remaining 4248 genes were ordered in terms of the time of their peak expression (Fig. 4b). As *P. falciparum* has an approximately 48 h intraerythrocytic cycle, to capture as many trophozoite-stage genes as possible, we considered the genes with a peak expression time point (***t***_**p**_) at 15–40 h to be highly expressed in the trophozoite stage [32]. Using this method, we identified a total of 3425 genes maximally expressed in this stage.

Comparing the 279 candidate genes with the genes expressed in the trophozoite stage, we obtained 55 candidate genes that overlapped between these groups (Fig. 4c and Additional file 11: Table S6). Most of them encode exported proteins and have never been studied, but several of them, such as PfEMP1 and RIFIN/STEVOR, have been reported to mediate the interaction between infected erythrocytes and endothelial cells [23, 33, 34]. Two genes were newly identified to contribute to CM, including genes encoding glycophorin binding protein (GBP) and parasite-infected erythrocyte surface protein 2 (PIESP2). The presence of the PEXEL motif and the transmembrane domains in these two proteins suggests their possible location on the surface of infected erythrocytes. The GBP protein contains a tandem repeat that can bind with glycophorin on the erythrocyte surface [35], implying that this protein might have a role in mediating the binding of infected erythrocytes to uninfected ones. PIESP2 is an erythrocyte surface protein and contains a gap-junction-related Neuromodulin_N domain in its extracellular region (Fig. 4d, upper panel). It was maximally transcribed at the trophozoite stage (***t***_p_ = 22.5 h, Fig. 4d, lower panel). In a serology study, antibody against PIESP2 in a malaria-protected group was much higher than that in a malaria-susceptible group [36], suggesting that the blockage of PIESP2 might confer a protective effect against malaria. In view of these features of PIESP2, we were prompted to consider that it might play a role in CM. Therefore, we selected this gene as an interesting target for further functional characterization in our lab.

## Discussion

In this study, to identify *P. falciparum* genes that contribute to human disease, we developed a virtual genome method that can be applied to identify genes enriched in a group of species, including group-specific genes and group-expansion genes. By this method, we looked for protein-coding genes in the *P. falciparum* genome that are responsible for parasitizing human erythrocytes, for human virulence, and for CM. Our method can be used not only for malaria genome comparisons, but also for other pathogen genome comparisons, such as for *Toxoplasma gondii* and *Mycobacterium tuberculosis*.

As mentioned previously, our method is much simpler and more comprehensive than previous comparative analysis methods; however, it has two limitations that should be pointed out. One limitation is that we used the modified BGLL algorithm to find disjointed clusters, but in practice many clusters overlap to some extent. Some vertices were shared by many clusters. Therefore, it is reasonable that an algorithm allowing cluster overlap should outperform the current BGLL method. Actually, we attempted to apply the extended Girvan and Newman algorithm and the clique percolation method to identify clusters overlapping within the protein similarity network [37, 38]. Owing to high requirements for computational resources and the analysis not being finished even 7 days after program initiation, we had to choose the fast-greedy method of using the BGLL algorithm instead. Another limitation is that edge weight was not considered when performing modularity analysis, leading to a failure to identify some clusters. For example, in the *P. falciparum* genome, the DBL family has three members, including genes encoding erythrocyte binding antigen-175 (EBA-175), EBA-140, and EBA-181 [39]. They were assigned with *var.* gene family members to Cluster_63, as all of these genes produce proteins containing DBL domains. Actually, despite significant similarities among these protein sequences, their alignment scores were quite different. The scores between any two members of the DBL family were much higher than those between members of the DBL family and members of PfEMP1. To overcome this issue, we can introduce edge weight, which represents the degree of conservation between the query and the target, to construct a weighted network. The identification of clusters within the weighted network might provide a better result.

We compared cluster values in human malaria parasites with those in rodent malaria parasites in the search for *P. falciparum* genes potentially responsible for parasitizing human erythrocytes. In total, 115 genes were identified to be enriched in human malaria parasites and to participate in many biological processes, such as thiamine biosynthesis, parasite growth and development, and purine metabolism. One peculiarity of human malaria parasites is that these species contain several genes whose proteins are involved in trafficking and the display of membrane proteins on the surface of infected erythrocytes, including three EMP1-trafficking protein-coding genes and *SURFIN4.2*. The disruption of some of these genes in *P. falciparum* resulted in a complete lack or greatly reduced expression levels of surface proteins on the surface of infected erythrocytes [19]. Thus, we proposed that human malaria parasites are capable of utilizing a distinctive transport system to export proteins on the membrane of infected erythrocytes. Additionally, 57 clusters that consist of 289 genes from *P. berghei* were enriched in rodent malaria parasites. Most of these genes are located within subtelomeric regions which usually contain various repeated elements. Subtelomeric regions are usually responsible for frequent duplication events and recombination events, which are mechanisms for generating antigenic diversity of genes and enhancing the adaption of organisms to the environment [40]. Cellular component analysis demonstrated that most of the proteins encoded by these genes are displayed on the surface of erythrocytes, and thus could be potential targets of the host’s immune response. In light of these results, we speculated that the majority of rodent parasite-enriched genes are probably involved in antigenic variation and immune evasion, subsequently contributing to survival in rodent erythrocytes and the establishment of long-lasting chronic infection, a process that is essential in malaria parasites to ensure mosquito transmission and the completion of the life cycle [41].

To search for genes related to the virulence of *P. falciparum* parasites, we compared enriched values of all clusters in *P. falciparum* with those in *P. vivax* and *P. knowlesi*. Finally, we identified 493 candidate genes. Some of these genes encode proteins related to cytoadhesion, such as RIFIN/STEVOR and PfEMP1 proteins. Others participate in erythrocyte invasion and the inhibition of antithrombin. In particular, a number of the *P. falciparum*-enriched genes were shown to be associated with membrane protein trafficking, including genes from the *FIKK*, *PfMC2TM*, *EPF3*, and *EPF4* families, suggesting that *P. falciparum* has a more powerful membrane protein transporting system than the other two human malaria parasites. One possible explanation for this is that the *P. falciparum* parasite has developed a unique cytoadhesion and antigenic variation system encoded by genes from *var.*, *rifin/stevor*, or other families. The trafficking and correct exposure of these molecules on infected erythrocytes require the assistance of a number of trafficking proteins encoded by the aforementioned genes. Therefore, these trafficking proteins could be novel therapeutic targets to reduce pathogen virulence by decreasing the exposure of virulence factors on the surface of erythrocytes.

In an attempt to discover novel genes that contribute to CM, by the integration of FFT analysis, we identified 55 candidate genes. Considering that surface antigen PIESP2 contains the gap-junction-related Neuromodulin_N domain and anti-PIESP2 might protect against malaria, we finally chose this protein as an interesting target for further experimental study. Supposed that PIESP2 participated in CM, blockage of this antigen by an antibody could be a promising strategy to prevent CM for the following reasons: One is that the *P. falciparum* parasite has an approximately 48-h intraerythrocytic developmental cycle and PIESP2 is highly expressed at the trophozoite stage, which means that the antibody against PIESP2 has more than 20 h to recognize and bind with this antigen before the release of new merozoites. Therefore, compared with antibodies against invasion-related parasite proteins, anti-PIESP2 might be more effective to prevent malaria infection because the parasite invasion process is extremely rapid (taking less than 2 min) and is only at risk of immunological attack for a very short time [42]. The other reason is that antibody against PIESP2 might disrupt the interaction between PIESP2 and its interactant on the endothelium surface, subsequently decreasing the binding of infected erythrocytes on the microvasculature. To date, we have successfully produced a soluble extracellular domain of PIESP2. Efforts to elucidate the function of PIESP2 are currently ongoing.

## Conclusions

In this study, to identify *P. falciparum* genes linked to human disease, we developed a new comparative analysis method that can be applied to find both group-specific and group-expansion genes. Through genome comparisons, we identified a limited number of genes in the *P. falciparum* genome related to parasitizing human erythrocytes, virulence, and CM. Our analysis not only revealed the genome-wide differences between *P. falciparum* and five other *Plasmodium* species, but also identified several novel genes that could serve as starting points for follow-up experimental investigations.

## Methods

### Protein sequence acquisition

Protein sequences of three human malaria parasites, *P. falciparum* 3D7, *P. knowlesi* strain H, and *P. vivax* Sal-1, and three rodent malaria parasites, *P. berghei* ANKA, *P. chabaudi* chabaudi, and *P. yoelii* yoelii 17XNL, were acquired from the database Plasmodb (http://plasmodb.org). Sequences with length ≤ 50 aa were removed. The remaining sequences were combined into a total set containing 5532 sequences from *P. falciparum*, 5320 sequences from *P. knowlesi*, 5580 sequences from *P. vivax*, 5070 sequences from *P. berghei* ANKA, 5211 sequences from *P. chabaudi*, and 6601 sequences from *P. yoelii*. The total set was used for sequence alignment.

### Protein sequence alignment

We employed phmmer instead of BLASTP to perform protein sequence alignment since it is more sensitive and accurate than BLASTP [43]. Thresholds ranging from 1E-01 to 1E-16 were tested. A hit with an expected value of less than the threshold was considered to be significant. By this rule, we established a protein correlation matrix**A =** [***a***_***ij***_]_**33314 × 33314**_, where *a*_*ij*_ = 1 indicates that protein *i* significantly hits protein *j*, and *a*_*ij*_ = 0 shows no significant hit between proteins *i* and *j*. We considered only the hits with mutual hits for two proteins in alignment analysis, that is,

$$ {\boldsymbol{a}}_{\boldsymbol{ij}}=\left\{\begin{array}{c}\mathbf{1}\kern0.75em \boldsymbol{if}\ {\boldsymbol{a}}_{\boldsymbol{ij}}={\boldsymbol{a}}_{\boldsymbol{ji}}=\mathbf{1}\kern9.5em \\ {}\mathbf{0}\kern0.5em \boldsymbol{if}\ {\boldsymbol{a}}_{\boldsymbol{ij}}={\boldsymbol{a}}_{\boldsymbol{ji}}=\mathbf{0}\ \boldsymbol{or}\kern0.75em {\boldsymbol{a}}_{\boldsymbol{ij}}\mathbf{\ne}\kern0.5em {\boldsymbol{a}}_{\boldsymbol{ji}}\kern2.75em \end{array}\right. $$

The obtained matrix was converted into a protein similarity network, which was composed of several separate components.

### Sequence cluster identification

We introduced a modularity method to find communities in the protein similarity network. Modularity refers to the fraction of the edges that fall within the given groups minus the expected value of such a fraction if the edges are distributed at random. It has been used to evaluate the cluster structure of networks from a global perspective. Despite the effectiveness of the modularity method in cluster identification, finding the maximum modularity involves NP-complete complexity [44] and exhibits a high computational consumption. The approximation method with the BGLL algorithm has been developed and widely used to find sequence clusters within a connected network [45]. BGLL algorithm consists of two steps: in the first step, each node is considered a cluster. A node is moved into the group of neighborhood nodes when the maximal modularity gain is positive. This process is applied to all nodes until the modularity value is not improved. In the second step, the clusters found in the first step is considered as nodes, and a new network is built. The edge weight between any two nodes is given by the sum of weights of edges in clusters. These two combined steps constituted a pass which is repeated until the maximum modularity is achieved. Here, to apply this algorithm to a disconnected network, we modified the BGLL algorithm in two aspects: First, the depth first search (DFS) algorithm was employed to extract all separate components; second, for the component with the number of nodes ≥3, the BGLL algorithm was recursively used until the modularity of the resulting subnetwork was below the cut-off value. We tested the cut-off values ranging from 0.1 to 0.5 to find reasonable clusters. For the component with the number of nodes ≤3, the BGLL algorithm was not applied, and the component was directly kept as a cluster. The modified BGLL algorithm was implemented in MATLAB 2015a.

### Homolog identification by profile hidden Markov model

The construction of a profile hidden Markov model (HMM) involves two steps: multiple sequence alignment and parameter estimation. Protein sequences in each of the designated clusters were subjected to multiple sequence alignment using MSAProbs [46]; then, the aligned ensembles were used to estimate the parameters of the profile HMM using HMMER3.1b1. The resulting models were searched against a reference proteome database to find possible homologs in genome-sequenced species though the web server HMMER (https://www.ebi.ac.uk/Tools/hmmer/search/hmmsearch). Bit score ≥ 40 was considered to be significant.

### Fast Fourier transform (FFT) analysis of gene expression data

RNA-seq sequence reads from eight time points of the intraerythrocytic cycle (GSE23787) were acquired [47]. Reads with low complexity, low quality, and multiple Ns were filtered out. Duplicated reads were also removed. Thereafter, the resulting clean reads were mapped against the *P. falciparum* genome (PlasmoDB v26) using HISAT2 [48]. The abundance of each reference gene was estimated with StringTie [49]. Relative transcriptional activity of each euchromatic gene was assessed using transcripts per million (TPM). When TPM < 1, it was adjusted to be 1. All expression values were log2-scaled and were used for FFT analysis.

FFT can be used to detect transcripts specific to a biological process, such as cell cycle and circadian clock. It converts an expression signal in the time domain to the frequency domain, showing the magnitude of each frequency [50]. The formula was as follows:

$$ {\boldsymbol{Y}}_{\boldsymbol{k}}=\frac{\mathbf{1}}{\sqrt{\boldsymbol{N}}}\sum \limits_{\boldsymbol{n}=\mathbf{0}}^{\boldsymbol{N}-\mathbf{1}}{\boldsymbol{X}}_{\boldsymbol{n}}{\boldsymbol{e}}^{-\boldsymbol{jk}\left(\frac{\mathbf{2}\boldsymbol{\pi }}{\boldsymbol{N}}\right)\boldsymbol{n}} $$

Here, *N* = 8 is the length of signal and *k* ≤ 7 is the frequency. The expression value of each transcript was centered by subtracting the mean value so that the amplitude equals zero at frequency *ω* = 0. Transcripts correlated with the cell cycle were selected as those whose maximal magnitudes *M* > 0.5 at frequency *ω* = 1. To estimate the maximal expression time point of selected transcripts, the phase value (*P*) at frequency *ω* = 1 was calculated. The maximal expression time point was estimated using the following formula:

$$ {\boldsymbol{t}}_{\boldsymbol{p}}=\left\{\begin{array}{c}\frac{\left(-\boldsymbol{P}\right)}{\mathbf{2}\boldsymbol{\pi }}\ast \mathbf{35}+\mathbf{5}\kern3em if\ P\  is\ negative\\ {}\frac{\left(-\boldsymbol{P}\right)}{\mathbf{2}\boldsymbol{\pi }}\ast \mathbf{35}+\mathbf{40}\kern2em if\ P\  is\ positive\end{array}\right. $$

Based on the ***t***_**p**_ value, we can identify genes highly expressed at a particular stage during the intraerythrocytic developmental cycle of malaria parasites.

### Enrichment analysis and sequence feature identification

To understand the biological meaning of a given gene set, we performed GO term enrichment analysis through the web server Plasmodb. A GO term was considered to be statistically overrepresented if its *p*-value was less than 0.05.

Protein sequence features, such as signal peptide and transmembrane domain, were analyzed using SignalP4.1 and TMHMM2.0 [51, 52], respectively. To avoid a signal peptide being wrongly predicted to be a transmembrane domain, the N-terminus of each sequence was truncated with a length of 25 aa. Tools were run with default parameters. PEXEL motif with the consensus R/KxLxE/Q is necessary for parasite protein export into the host erythrocytes [15]. Proteins containing this motif were identified via the web server Plasmodb.

## Additional files

Additional file 1: **Table S1.** The corresponding relationships between 33,314 genes and 4605 clusters. (XLSX 605 kb)

Additional file 2: **Figure S1.** Clusters composed of members from a single species or six species. **a**) Clusters comprise *P. vavix* genes (left panel) or *P. falciparum* genes (right panel). **b**) Clusters comprising genes from six *Plasmodium* species. (TIF 1617 kb)

Additional file 3: **Table S2.** Enriched values of all clusters in six *Plasmodium* species. (XLSX 154 kb)

Additional file 4: **Table S3.** The candidate *P. falciparum* genes probably responsible for parasitizing human erythrocytes. (XLSX 16 kb)

Additional file 5: **Table S4.** The *P. berghei* genes included in rodent malaria parasite-enriched clusters. (XLSX 20 kb)

Additional file 6: **Figure S2.** Genomic location of 115 *P. falciparum* genes. (TIF 968 kb)

Additional file 7: **Figure S3.** Genomic location of 267 *P. berghei* genes. (TIF 2034 kb)

Additional file 8: **Figure S4.** Expression dynamics of *SURF* family members in the intraerythrocytic cycle of the *P. falciparum* parasite. (TIF 75 kb)

Additional file 9: **Table S5.** Candidate genes related to virulence of the *P. falciparum* parasite. (XLSX 31 kb)

Additional file 10: **Figure S5.** Conserved peptide region identified in PfEMP1 variants. Upper panel, multiple sequence alignment of conserved regions from PfEMP1 proteins. Lower panel, sequence logo showing the conserved peptide region. (TIF 1250 kb)

Additional file 11: **Table S6.** Identified *P. falciparum* genes that possibly contribute to cerebral malaria. (XLSX 12 kb)

## Abbreviations

ATS: Acidic terminal segment
CIDR: Cysteine-rich inter-domain region
CM: Cerebral malaria
DBL: Duffy-binding-like
EBA-175: Erythrocyte binding antigen-175
EPCR: Endothelial protein C receptor
EPF3: Exported protein family 3
GBP: Glycophorin binding protein
GO: Gene Ontology
HRPII: Histidine-rich protein II
ICAM1: Intercellular adhesion molecule 1
MC2TM: Maurer’s cleft two transmembrane
MSP2: Merozoite surface protein 2
PCA: Principal component analysis
PfEMP1: *P. falciparum* erythrocyte membrane protein 1
PHISTa: *Plasmodium* helical interspersed subtelomeric family subtype a
PIESP2: Parasite-infected erythrocyte surface protein 2
PIR: Plasmodium interspersed repeat
RBC: Red blood cell
SICA_C: Schizont-infected cell agglutination C-terminal
