# Supplementary figures and images for: In-depth comparative analysis of malaria parasite genomes reveals protein-coding genes linked to human disease in Plasmodium falciparum genome
Source: BMC Genomics. 2018 May 2;19:312. doi: 10.1186/s12864-018-4654-5 (PMC5930813; doi:10.1186/s12864-018-4654-5)

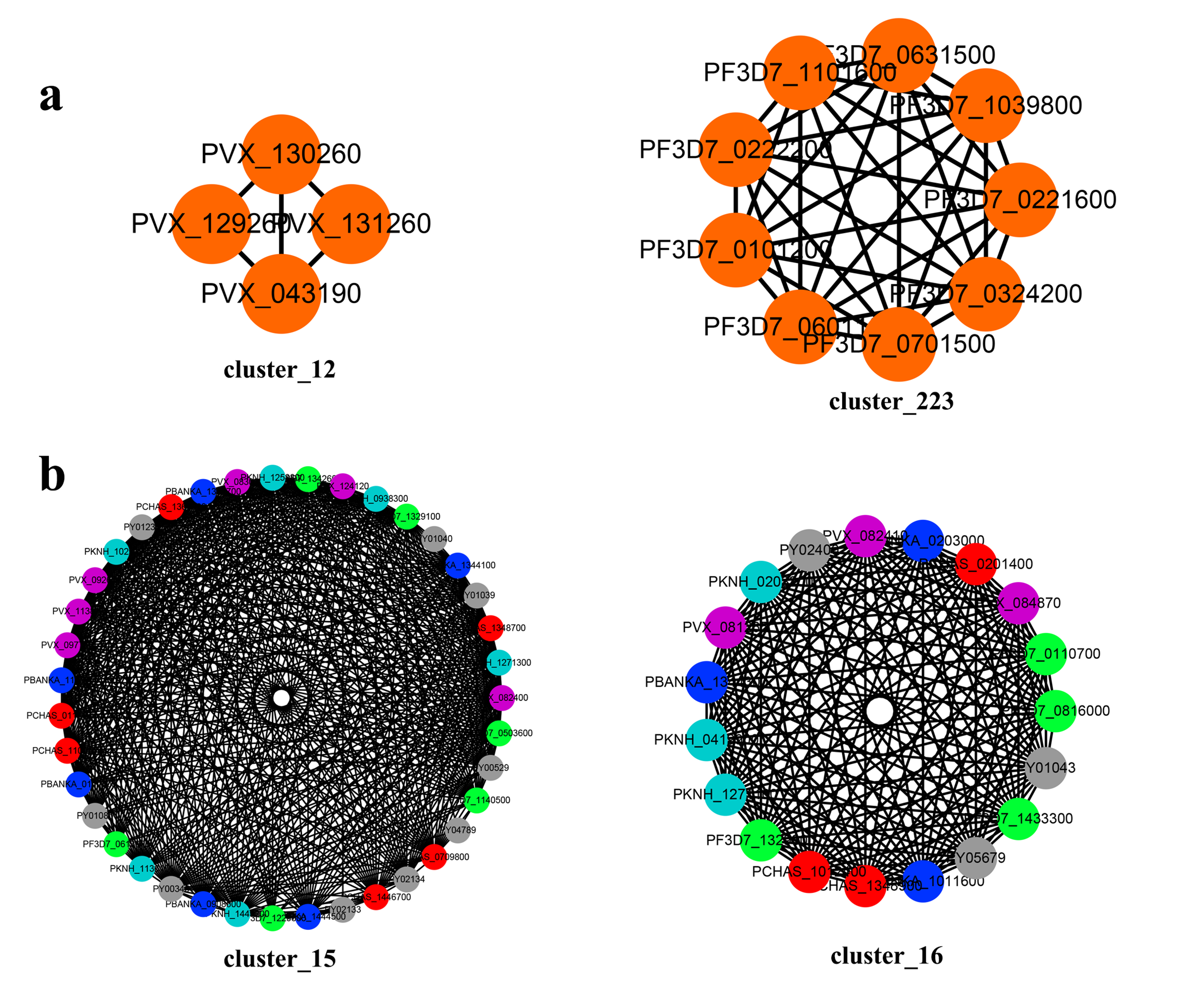

Supplement: Supplementary file 2 — Figure S1. Clusters composed of members from a single species or six species. a) Clusters comprise P. vavix genes (left panel) or P. falciparum genes (right panel). b) Clusters comprising genes from six Plasmodium species. (TIF 1617 kb) [file 12864_2018_4654_MOESM2_ESM.tif]

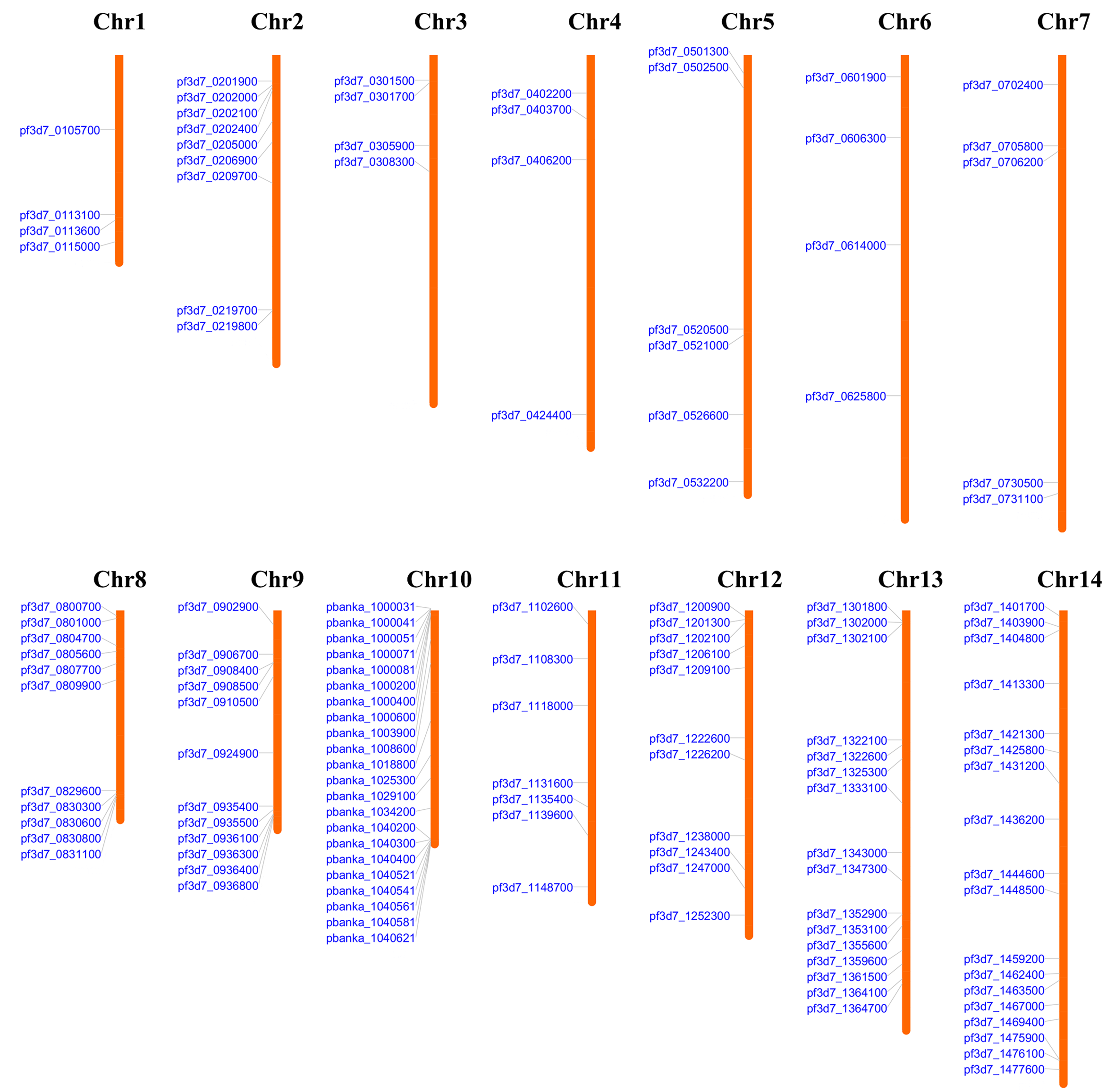

Supplement: Supplementary file 6 — Figure S2. Genomic location of 115 P. falciparum genes. (TIF 968 kb) [file 12864_2018_4654_MOESM6_ESM.tif]

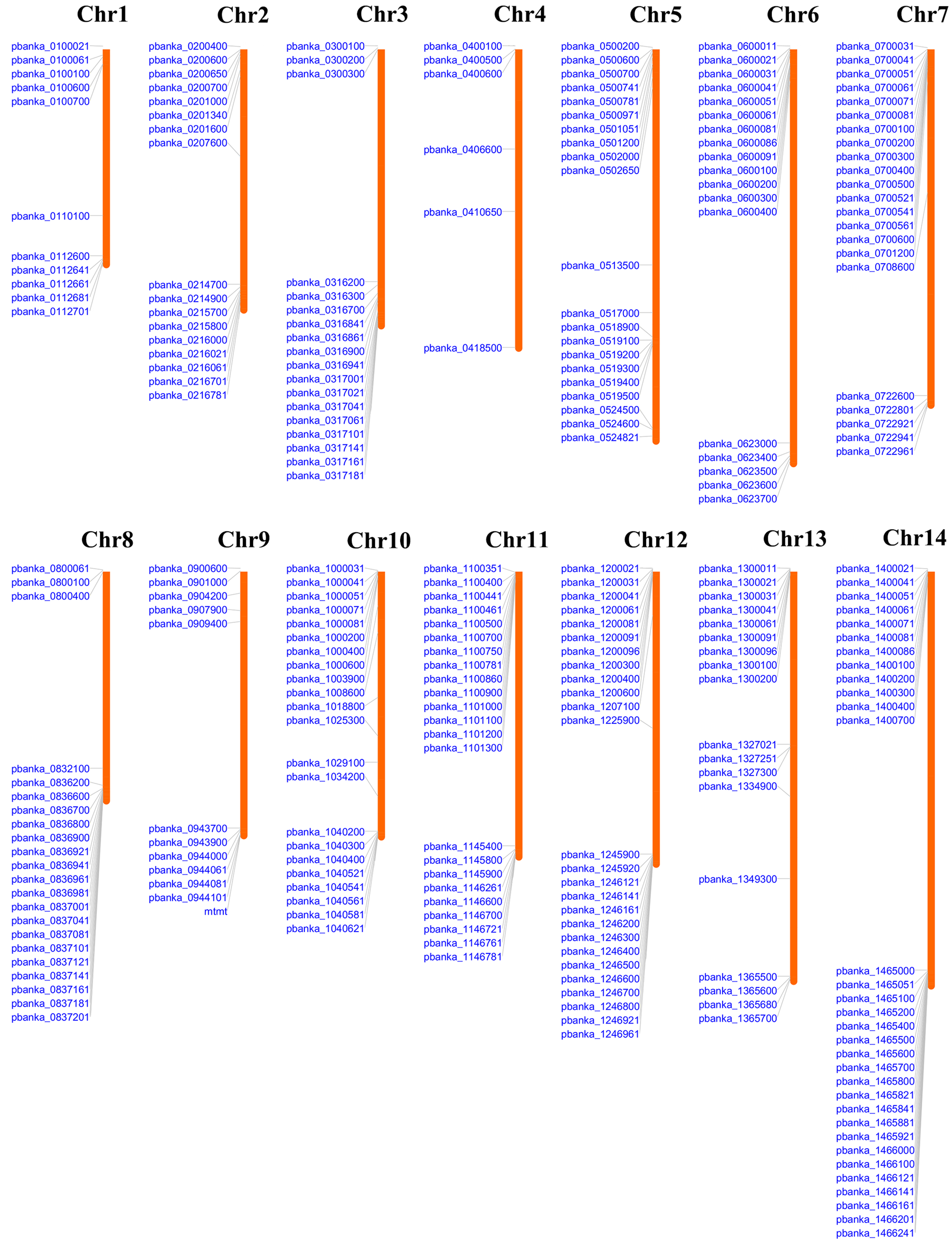

Supplement: Supplementary file 7 — Figure S3. Genomic location of 267 P. berghei genes. (TIF 2034 kb) [file 12864_2018_4654_MOESM7_ESM.tif]

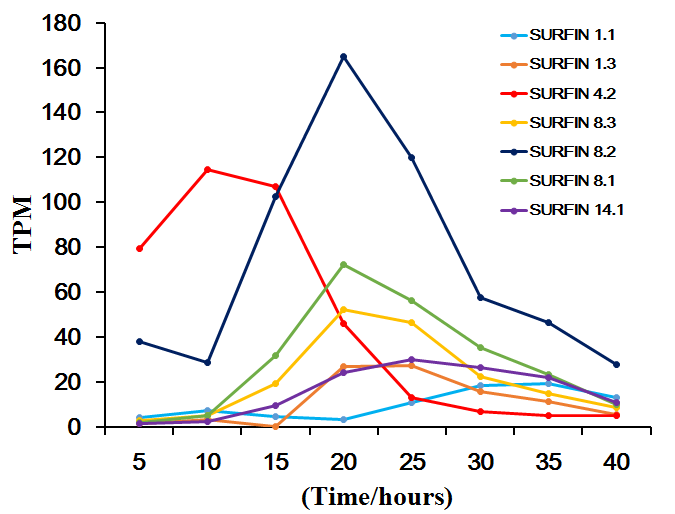

Supplement: Supplementary file 8 — Figure S4. Expression dynamics of SURF family members in the intraerythrocytic cycle of the P. falciparum parasite. (TIF 75 kb) [file 12864_2018_4654_MOESM8_ESM.tif]

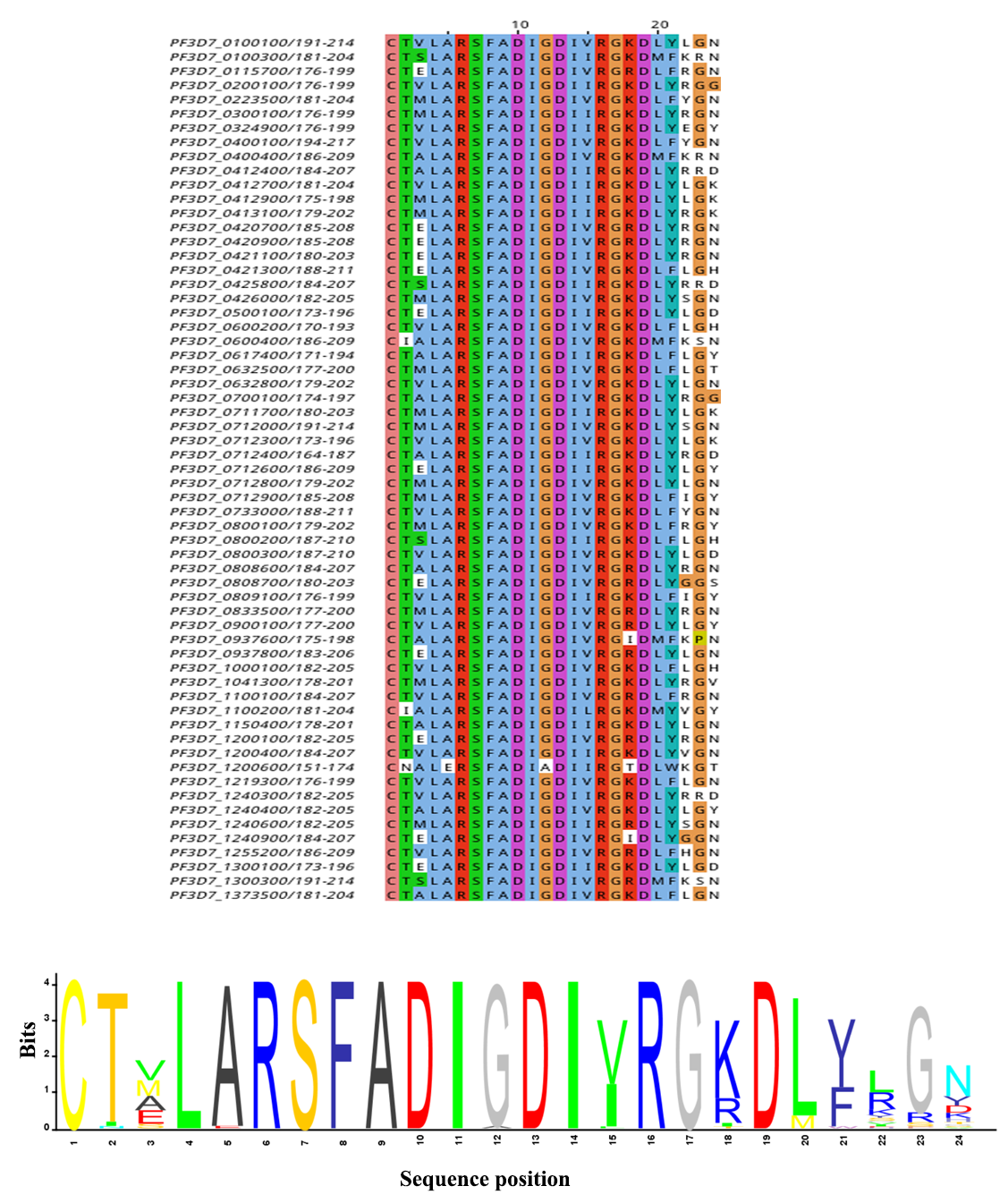

Supplement: Supplementary file 10 — Figure S5. Conserved peptide region identified in PfEMP1 variants. Upper panel, multiple sequence alignment of conserved regions from PfEMP1 proteins. Lower panel, sequence logo showing the conserved peptide region. (TIF 1250 kb) [file 12864_2018_4654_MOESM10_ESM.tif]
